# New Trends in Shoulder Surgery from Biomechanics to the Operating Room

**DOI:** 10.3390/jcm10112498

**Published:** 2021-06-05

**Authors:** Edoardo Franceschetti, Edoardo Giovannetti de Sanctis, Giovanni Di Giacomo, Francesco Franceschi

**Affiliations:** 1 Department of Orthopaedic and Trauma Surgery, Campus Bio-Medico University of Rome, Via Alvaro del Portillo 200, 00100 Rome, Italy; 2 Department of Orthopaedics and Traumatology, Fondazione Policlinico Universitario Agostino Gemelli IRCCS, Piazzale Agostino Gemelli 8, 00168 Rome, Italy; edoardo.giovannettids@gmail.com; 3 Department of Orthopaedic and Trauma Surgery, Concordia Hospital for Special Surgery, 00145 Rome, Italy; concordia@iol.it; 4 Department of Orthopaedic and Trauma Surgery, San Pietro Fatebenefratelli Hospital, Via Cassia 600, 00189 Rome, Italy; f.franceschi@unicampus.it

## 1. Introduction

After a rigorous peer review process, twelve papers were accepted and published. These papers cover a wide range of topics including shoulder arthroscopy, arthroplasty and related neuropathies.

This Special Issue focuses on “New trends in shoulder surgery from Biomechanics to the operating room”, and aims to cover recent advancements as well as future trends in the field of shoulder surgical treatments. 

Lee et al. [1] evaluated the functional and radiologic outcomes of biocompatible non-absorbable PEEK and biocomposite anchors used during rotator cuff repair, focusing on the rate of perianchor cyst formation during the first six months postoperatively. This paper demonstrated that the biocomposite anchor has a statistically significant tendency to form higher grades of fluid collection at 3 months after surgery, though this is usually reduced by the sixth postoperative month.

Three papers published by the group of Romano et al. [2,3,4] are focused on both shoulder arthroscopy and arthroplasty.

The first one [4] proves that the Infinity-lock button system is effective for the treatment of chronic grade III AC joint dislocation, resulting in elevated satisfaction ratings and predictable outcomes.

The second paper [3] analyzes midterm results of onlay lateralized cementless stem reverse total shoulder arthroplasty in patients with valgus/varus malunion proximal humerus fracture sequelae without metaphyseal osteotomy, showing improved clinical outcomes and decreased complications.

Our interest in this new prosthesis design has increased over the last decade too, showing better clinical outcomes while using an onlay curved lateralized cementless stem [5,6].

The third paper [2] emphasizes the use of MicroDTTect, bioactive glasses and tantalum cones in periprosthetic joint infections and revision surgery of the shoulder. The application of those instruments would allow for earlier bacterial identification and higher prosthesis–bone interface stability, respectively.

Therefore, we are also proud to present the results of our study on the efficacy of the lateral acromioplasty [7]. This technique seems to be safe and reproducible, preventing the recurrence of rotator cuff tears in patients with small and medium lesions and a CSA greater than 35*. A mathematical formula has been proposed to resect the correct amount of acromial bone and decrease the CSA within a favorable range (30°–35°).

Lädermann et al. [8] compared scapulothoracic alignment in pathologic type B shoulders with contralateral healthy shoulders. The non-statistically significant results of this study found that Walch type B shoulders had some limitations in maximal glenohumeral motion but exhibited significantly greater anterior scapular tilt with internal rotation, which might be adaptive.

Therefore, Lädermann et al. [9] have published an observational study with the aim of determining the incidence of OBPP (obstretical brachial plexus palsy) risk factors in type B patients. They concluded that perinatal factors related to OBPP did not occur in a higher frequency in patients with Walch type B OA compared to the general population, although some of them were in the high normal range.

The study presented by Riedl et al. [10] showed the first clinical and structural results of the recently introduced loop LHB tenodesis procedure. This technique provided good-to-excellent overall clinical results after a short-term follow-up of six months, with an inferior incidence of cosmetic deformities compared to conventional therapy options, such as tenotomy and anchor tenodesis.

The proper treatment for the long head of the biceps is a topic still debated. Treatments available range from tenotomy to tenodesis, which has been described using different systems and in different positions along the humerus. Our group has recently published a paper comparing high and subpectoral tenodesis during rotator cuff repair [11].

Pascarella et al. [12] tested and demonstrated the effectiveness of triple monitoring (a combination of ultrasound, nerve stimulation and opening injection pressure) while performing an interscalene brachial plexus block (IBPB) for arthroscopic shoulder surgery.

Three review papers complete this Special Issue.

Bozzi et al. [13], with their “Suprascapular Neuropathy around the Shoulder: A Current Concept Review”, present an overview of the state-of-the-art diagnosis and treatment of suprascapular neuropathy. Nerve entrapment is an uncommon but increasingly recognized cause of shoulder pain. It is more frequently diagnosed in over-head athletes or patients with massive rotator cuff tears. Prompt diagnosis is crucial to treat it successfully through a conservative or an arthroscopic procedure, which is then considered the gold standard.

Yon CJ et al. demonstrate that arthroscopic revision Bankart repair might lead to an improvement in clinical outcomes and reasonable satisfaction with proper patient selection [14].

Giovannetti de Sanctis et al. [15] compare corticosteroid injections to other drugs in the treatment of partial rotator cuff tears, focusing on the effectiveness of this therapeutic modality in terms of pain and shoulder functionality. PRP injections seem to lead to significantly better outcomes in terms of pain and shoulder function in long-term follow-up.

Given these different contributions, it is evident that shoulder surgery still has fundamental questions that remain unanswered.

We would like to thank the Editor in Chief for his support throughout the preparation of this Special Issue. We are grateful to all the anonymous reviewers who devoted their precious time reviewing the papers submitted to this Special Issue. Their reviews helped us select the best papers to be included in this Special Issue. We would like also to thank all authors who contributed to this Special Issue.

Hopefully you will enjoy reading this selection of articles, as we did, and you will find them informative and helpful in keeping yourself up to date in the field of shoulder surgery.

## Data Availability

Not applicable.
